# A Mechanistic Individual-Based Model of the Feeding Processes for *Oikopleura dioica*

**DOI:** 10.1371/journal.pone.0078255

**Published:** 2013-11-05

**Authors:** Maxime Vaugeois, Frédéric Diaz, François Carlotti

**Affiliations:** 1 Aix Marseille Université, French National Centre for Scientific Research/INSU, IRD, Mediterranean Institute of Oceanography (MIO), UM 110, Marseille, France; 2 Université de Toulon, CNRS/INSU, IRD, Mediterranean Institute of Oceanography, UM 110, La Garde, France; National Science and Technology Development Agency, Thailand

## Abstract

A mechanistic physiological model of the appendicularian *Oikopleura dioica* has been built to represent its three feeding processes (filtration, ingestion and assimilation). The mathematical formulation of these processes is based on laboratory observations from the literature, and tests different hypotheses. This model accounts for house formation dynamics, the food storage capacity of the house and the gut throughput dynamics. The half-saturation coefficient for ingestion resulting from model simulations is approximately 28 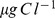 and is independent of the weight of the organism. The maximum food intake for ingestion is also a property of the model and depends on the weight of the organism. Both are in accordance with data from the literature. The model also provides a realistic representation of carbon accumulation within the house. The modelled half-saturation coefficient for assimilation is approximately 15 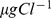 and is also independent of the weight of the organism. Modelled gut throughput dynamics are based on faecal pellet formation by gut compaction. Model outputs showed that below a food concentration of 30 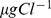, the faecal pellet weight should represent a lower proportion of the body weight of the organism, meaning that the faecal pellet formation is not driven by gut filling. Simulations using fluctuating environmental food availability show that food depletion is not immediately experienced by the organism but that it occurs after a lag time because of house and gut buffering abilities. This lag time duration lasts at least 30 minutes and can reach more than 2 hours, depending on when the food depletion occurs during the house lifespan.

## Introduction

In pelagic ecosystems, appendicularians are thought to play a significant role in both the carbon cycle and ecosystem structuration [1], [2]. Two features led to this assumption: first, appendicularians are circumglobal distributed organisms and are among the dominant mesozooplankton group in many marine ecosystems [3]. Second, their specific biology allows them to occupy a unique ecological niche in the food web.

Appendicularians live inside a self-produced structure called a “house” that can be viewed as a micro-environment controlled by the organismal processes of water pumping and filtration; by beating its tail, the organism creates a water flow that streams particles from the outside to the inside of the house [4], [5], allowing for quantitative control; on the other hand, qualitative control is carried out by a complex system of filters (three filters with different mesh sizes), which also enables the organism to select particles across a large size-prey spectrum [6]. Consequently, the length ratio between an appendicularian and its prey may reach 1∶10,000 [1] because they are able to shortcut the microbial loop and to feed on pico and nano phytoplankton. Furthermore, because appendicularians are an important source of food for teleosts [7], they represent a shorter trophic pathway from the small primary producers to the higher trophic levels [1], [8]. Their ability to feed on both very small-sized organisms (bacteria or pico and nano phytoplankton) and on larger organisms (micro phytoplankton, nano and micro zooplankton) also gives them an advantage in terms of food supply but requires a significant energy cost in return. In particular, the house must be replaced several times during the organism’s life, and the weight of each house represents a substantial proportion of the organism’s body weight [5], [9], [10]. Many houses and their detritus are released into the environment during the organismal life cycle, which can make a major contribution to marine snow [11], [12]. Moreover, because of their high filtration rate [5], [6], appendicularians produce a large quantity of faecal pellets [1], [6] that contribute to carbon export. This large production of sinking discarded houses and faecal pellets [9], [11], [13] can represent a strong carbon flux because appendicularians can be present at high densities (up to 53,000 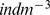 for *Oikopleura dioica* [14]; up to 3,565,000 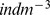 for *Oikopleura longicauda* [3]; up to several hundred 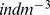 for *Oikopleura vanhoeffeni* [15]).

Appendicularians were first described by Chamisso and Eysenhart in 1821 (cited *in* [5]), and more than 70 species distributed among 3 families (*Oikopleuridae, Fritillaridae* and *Kowalevskiidae*) are known [3]. Among all of the appendicularian species, *Oikopleura dioica* has been the most studied. Indeed, the ability to cultivate this species under laboratory conditions has been developed over the last 40 years [16]–[18], which has enabled numerous scientific studies. Some experiments have focussed on one or more food supply processes and/or organic matter production (house or faecal pellet production) in *Oikopleura dioica*. From those experimental results, different empirical to mechanistic models have been proposed. To our knowledge, four models focussed on feeding processes (filtration, ingestion and/or assimilation) have been announced. Fernandez *et al.* [19] showed that filtration and ingestion depend on both the particle size and the organism size. Based on their experiments, they developed an empirical model that accurately reproduces actual data. Nevertheless, the proposed model does not represent the assimilation process, as this study was focussed on filtration and ingestion processes. López-Urrutia *et al.* [20] proposed an individual growth model to enable the estimation of food-limited growth conditions. However, the filtration and egestion processes were not explicitly represented, and a constant assimilation rate was assumed. Consequently, the López-Urrutia *et al.* model cannot represent either the amount of carbon trapped within the houses and released throughout the appendicularian life or the faecal pellet production. Moreover, experimental observations demonstrated that the assimilation rate varies with the food concentration [13], [21]. A model proposed by Lombard *et al.* [22] represents the different steps of the feeding process as well as processes involved in the growth balance. With regards to these different steps, ingestion and assimilation are forced by the ingestion and assimilation efficiency functions, respectively. Consequently, this model cannot explain the ingestion and assimilation response to the quantity of available substrate, *i.e.*, the food quantity trapped within the house for the ingestion process and the gut content for the assimilation process. Nevertheless, the Lombard *et al.* model is able to calculate the total amount of carbon trapped within the houses and the amount of carbon released as faecal pellets into the environment. However, it does not explicitly represent the house effect because the ingestion process is formulated depending on the environmental food concentration; moreover, faecal pellet production is represented as a continuous process, whereas it is actually a discrete process [13]. Finally, the model proposed by Touratier *et al.* [23] is the most mechanistic compared to the previous models. This model simulates food concentration dynamics within the house as a balance of external particle filtration and the ingestion of particles trapped in the house. Moreover, the Touratier *et al.* model accounts for the house replacement effect. Nonetheless, assimilation efficiency is treated as a constant, and faecal pellet production is represented as a continuous process.

The present study proposes a mechanistic model that focusses on all three processes linked to carbon energy uptake in the appendicularian species *Oikopleura dioica*. It does not take into account the effect of particle size on the filtration, ingestion and assimilation processes because it is calibrated with experimental data from a similar mono-algal diet (*i.e.*, same particle size and same carbon mass per cell). The model concept is based on published experimental observations and will be used to test different hypotheses on the physiology of this organism. Moreover, our model is built to simulate the carbon mass dynamics of the house and gut contents, and it formulates the ingestion and assimilation processes as depending on them for a better understanding of their dynamics. This model accounts for the house replacement effect and simulates faecal pellet production as a discrete process. We also use simulations of starvation to investigate the house and gut ability to smooth fluctuations in environmental food availability.

## Methods

### 1 The Model

#### 1.1 Model structure

**Figure 1:**
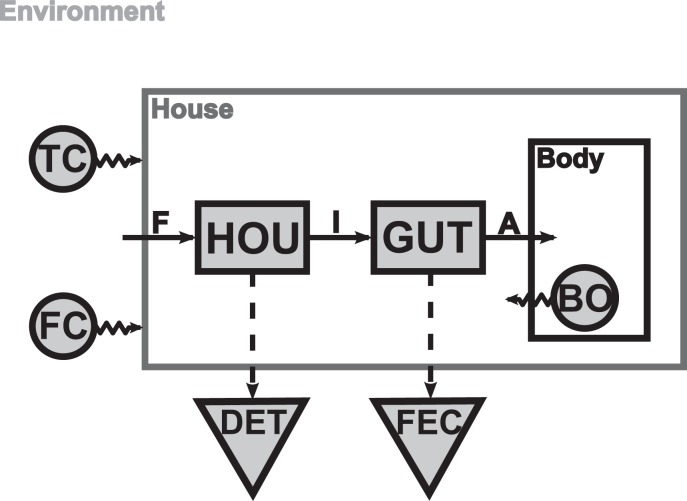
Conceptual schema of the model. Forcing variables are represented by round boxes, state variables by rectangular boxes and diagnostic variables by triangular boxes. Solid arrows represent continuous fluxes and dashed arrows represent discontinuous fluxes. FC: environmental food concentration (in 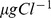), TC: environmental temperature (in degree Celsius), BO: carbon mass of the organism’s body (in 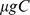), HOU: carbon mass of the house contents (in 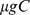), GUT: carbon mass of the gut contents (in 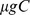), DET: accumulated carbon mass trapped within all the houses produced and released along the appendicularian life (in 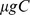), FEC: accumulated mass of unassimilated carbon (*i.e.*, faecal pellets) released into the environment (in 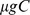), F: Filtration (in 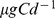), I: Ingestion (in 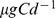), A: Assimilation (in 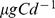).

The model (figure 1) focusses on energy intake processes from the environment, and it has been built using three different types of variables, namely, three forcing variables, two state variables and two diagnostic variables.

The temperature in degrees Celsius (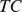) and the environmental food concentration (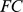) are the two environmental forcing variables, whereas the organismal body weight (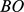) is an internal forcing variable. The system state is represented by two state variables, namely 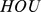, which represents the carbon mass of the appendicularian house contents, and 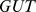, which represents the carbon mass of the gut contents. Finally, we used the following two diagnostic variables: the accumulated carbon mass trapped within all the houses produced and released over the appendicularian’s life (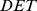), and the accumulated mass of unassimilated carbon (*i.e*, faecal pellets) released into the environment (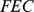).

#### 1.2 Modelled processes

The feeding process is a succession of three processes, namely, filtration, ingestion and assimilation. All these processes are accomplished by different mechanical structures within the organism. Therefore, even if the intensity of a process depends on the intensity of the preceding process, it remains independent with respect to implementation. As a consequence, there is a carbon loss between every step of the feeding process. The model was built to account for this independence from a mechanical standpoint, but it retains the dependence of each process on the intensity of its preceding process.

Food first enters the house through the filtration process, which is known to be influenced by numerous factors: first, by the individual body weight of the organism [24]–[27]; second, by the environmental temperature [28]; and last, by food concentration [27], [29], [30]. Therefore, we chose the following filtration function form, which was previously applied to *Oikopleura dioica* by Lombard *et al.* [22], to represent the filtration process in our model:
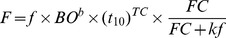
(1)

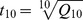
(2)This filtration function (F) is expressed in micrograms of carbon per day (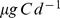), and 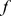 is the maximum filtration rate at 0°C This rate is influenced by the body weight of the organism through a power function (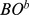), by the environmental temperature through the exponential function with a base 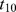 (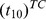) and by the food concentration through a Michaelis-Menten function with a half-saturation constant (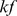).

Afterwards, part of the food accumulated within the house is ingested by the organism. This action is the ingestion process, which enables carbon to pass inside the gut, increasing its content (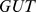). To represent this process, we propose the following ingestion function (I):
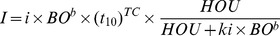
(3)Our formulation of the ingestion process is based on two experimental observations. First, the ingestion process is similarly influenced by the environmental temperature and the body weight of the organism as the filtration process [15], [31], [32]. Furthermore, the ingestion process depends on the environmental food condition [27], *i.e.*, the food quantity contained inside the house. Therefore, we propose an ingestion function that depends on the carbon mass of the house content through a Michaelis-Menten relationship. The maximum ingestion rate coefficient (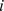) depends on the individual body weight and temperature, similarly to the filtration function. Moreover, the half-saturation coefficient (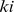) depends on the weight of the organism by the same power function that influences the maximum rates of filtration and ingestion. This new formulation of the half-saturation coefficient provides an equivalent ingestion efficiency (the amount of ingested carbon over the amount of filtered carbon) for any organism of any weight (see appendix S1). At any time, the house is filled by the filtration process and is emptied by the ingestion process (equation 1, appendix S1). Discontinuously, *Oikopleura dioica* leaves its house for a new one. Because each new house is initially empty, the house content variable (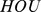) is reset to zero. Biologically, the reset frequency time, *i.e.*, the house renewal rate, primarily depends on the temperature [10], [33]. We defined the reset frequency time of the model using the house renewal rate function (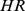), which was elaborated at a salinity of 30 by Sato *et al.* [10]:



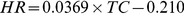
(4)The last step of the feeding process is assimilation: part of the carbon gut content is assimilated and will be available for the growth, reproduction and maintenance of the organism. To represent the assimilation process in our model, we propose the following assimilation function (A):
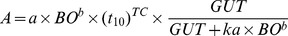
(5)Because the assimilation process is the least documented process in the literature, we based its formulation on three assumptions. First, we assume that this process depends on the environmental food condition [13], [21], *i.e.*, the quantity of food contained inside the gut, with a Michaelis-Menten relationship. Then, we assume that the assimilation process depends on the environmental temperature and on the individual body weight, similarly to the filtration and ingestion processes. Moreover, assuming that the assimilation efficiency (the amount of assimilated carbon over the amount of ingested carbon) does not depend on the weight of the organism, we use the same formulation of the half-saturation coefficient as the one described above for the ingestion process. At any given time, the gut is filled by the ingestion process and emptied by the assimilation process (equation 2, appendix S1). It also empties itself discontinuously by the egestion process, *i.e.*, by faecal pellet production. When the gut content reaches a maximum value, it empties itself by a value corresponding to the weight of a faecal pellet (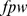, equation 6). Each faecal pellet represents a constant part (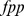) of the total organism weight (based on [34], *in* [22]). Here, we applied the same organism body weight dependence that is used for assimilation to the faecal pellet weight to ensure a strict independence of the assimilation efficiency from the body weight of the organism. With regards to the maximum value of the gut content (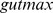, equation 7), we assume that its value is equal to the weight of a faecal pellet (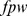, equation 6) multiplied by the mean number of faecal pellets in the gut (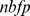, from [13]).



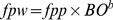
(6)

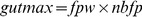
(7)


### 2 Body Weight and Size

In the present model, the body weight represents the weight of the entire organism, including the trunk and the gonad. We used the body length (including the trunk and gonad) to body weight function developed by Lombard *et al.* [35]. When we estimated quantities from the literature and referred it to a carbon body weight, the calculation was carried out by converting the body length of the organism to body weight using this equation. When needed, we converted the trunk length to the total body length using the Acuña *et al.*’s [27] function.

### 3 Parameter Estimation

The model structure and mathematical formulations of the different processes provide a qualitative behaviour close to the experimental observations. To ensure a quantitatively good simulation of experimental data, we estimated four of the ten model parameters (table 1). The filtration parameters (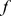 and 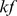) were not estimated because we decided to extract the filtration function and all its parameter values from Lombard *et al.* [22]. In that study, an estimation of those parameters accounting for the relationship between the body weight function (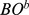 ) and the temperature function (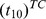) was already performed. The parameters concerning the faecal pellet weight and the maximum gut content value (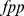 and 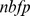) were also extracted from the literature.

**Table 1:** Model parameters.

Symbols	Description	Units	Values	Source
*b*	Exponent of the allometric equationfor filtration at 0°C	wd	0.9	[22]
*t*_10_	10th root of the Q10 coefficientestimated for filtration	wd	1.06	[22]
*f*	Maximum food intake for filtration at 0°C	*μgCμgC* ^–*b*^ *d* ^–1^	3.7	[22]
*kf*	Half-saturation constant for filtration	*μgCl* ^–1^	150	[22]
*i*	Maximum food intake for ingestion at 0°C	*μgCμgC* ^–*b*^ *d* ^–1^	1.2232	estimated
*ki*	Half-saturation constant for ingestion	*μgCμgC* ^–*b*^	0.0282	estimated
*a*	Maximum food intake for assimilation at 0°C	*μgCμgC* ^–*b*^ *d* ^–1^	0.4818	estimated
*ka*	Half-saturation constant for assimilation	*μgCμgC* ^–*b*^	0.0029	estimated
*fpp*	Faecal pellet size proportion	*μgCμgC* ^–*b*^	0.0175	[22]
*nbfp*	Mean number of faecal pellets in the gut	wd	3.368	[13]

To perform this parameter estimation, we first chose the data set with which we wanted to adjust the model simulations. We chose six sets of data extracted from three different experimental studies (table 2) in which the food quality conditions were the same (*i.e.*, same particle size and same carbon mass per cell). We then used the least squares method, which consists of finding a solution that minimises the sum of the squares of the errors to find the best set of parameters. To approximate this solution, we used a hybrid method of optimisation that combines the simulated annealing (SANN, [36]) and the limited-memory Broyden-Fletcher-Goldfarb-Shanno (L-BFGS-B, [37]) methods. Simulated annealing is a stochastic global optimisation method. It is frequently used for the global optimisation problem of locating a good approximation to the global optimum of a given function in a large search space. The L-BFGS-B method uses a limited-memory modification of the BFGS quasi-Newton method, which is used for solving non-linear optimisation problems. For each set of parameters and for each data set, the model was run over four house cycles (almost twelve hours) at the corresponding experimental temperature and food concentration. A house cycle starts when one house is deployed and ends when the next house is deployed. To obtain a realistic set of parameters, the space of the parameter values was bounded, when possible, with published data, and the assimilation parameters were set lower than the ingestion parameters.

**Table 2:** Experimental conditions of experiences from which data were used for parameter estimation.

Study	Temperature	Food Quality	Extracted Data
[35]	15°C	*Thalassiosira pseudonana* (4 to 5 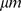 ESD, 8.8 to 13.3 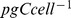 )	Figures 5a and 5c
[27]	15°C	*Isochrysis galbana* (5.5 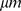 ESD, 10.6 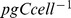 )	Figure 5d
[29]	20°C	*Isochrysis galbana* (4.1 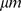 ESD, 10.6 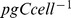 )	Figure 5b

ESD is the equivalent spherical diameter.

The L-BFGS-B method provides an estimation of the Hessian matrix. From this matrix, we calculated the covariance matrix and used it to generate random sets of parameters with multivariate normal distribution. This calculation allows us to explore a space of parameter values in which the function cost value does not change excessively (mean 7.12%). The 95% confidence interval of the estimated parameters is presented in table 3.

**Table 3:** 95% confidence interval of the optimised set of parameters.

Symbols	Values	Lower bound	Upper bound
*i*	1.2232	1.2229	1.234
*ki*	0.0282	0.0281	0.0284
*a*	0.4818	0.4815	0.4822
*ka*	0.0029	0.0026	0.0032

### 4 Simulations and Analytical Approach to Model Outputs

Several simulations were run to test the model behaviour using initial values of zero for 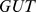 and 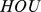, yielding the following: [i].

**Figure 2:**
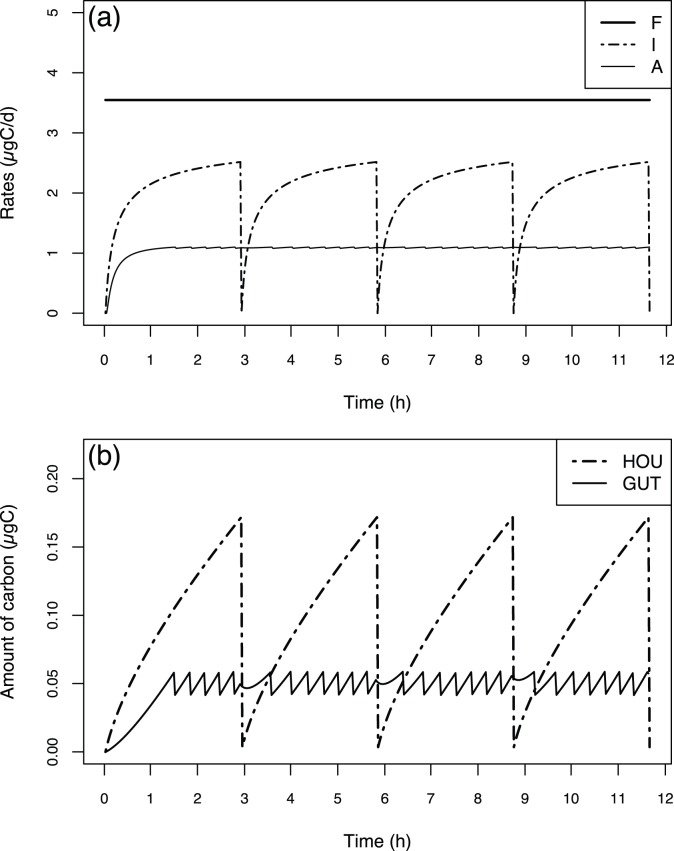
Fluxes and state variables dynamics during simulation at constant food concentration. Rates of filtration (F), ingestion (I) and assimilation (A) in 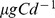 (a); carbon mass of the house contents (HOU) and carbon mass of the gut contents (GUT) (b) in 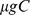. Run performed at a temperature of 15°C and a food concentration of 100 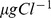 over four house cycles for an individual organism of 1 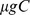.

**Figure 3:**
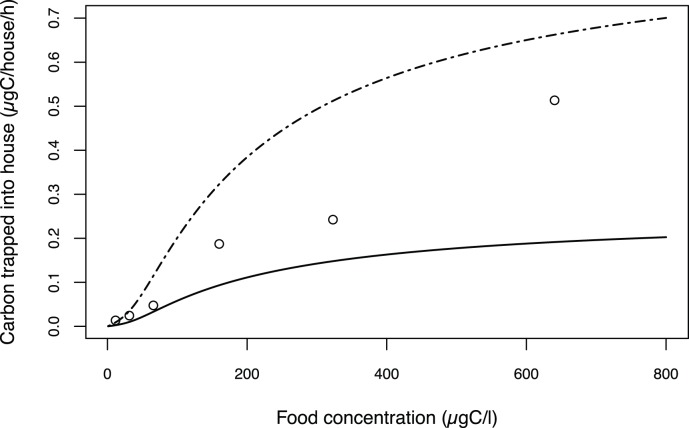
Simulated carbon accumulation rate within a house as function of the environmental food concentration (from 0 to 800 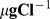). Simulations for an organism weight of 1 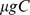 (solid line), and an organism weight of 3.97 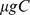 (dashed line) versus experimental data from Acuña *et al.* [27] (empty circles).

**Figure 4:**
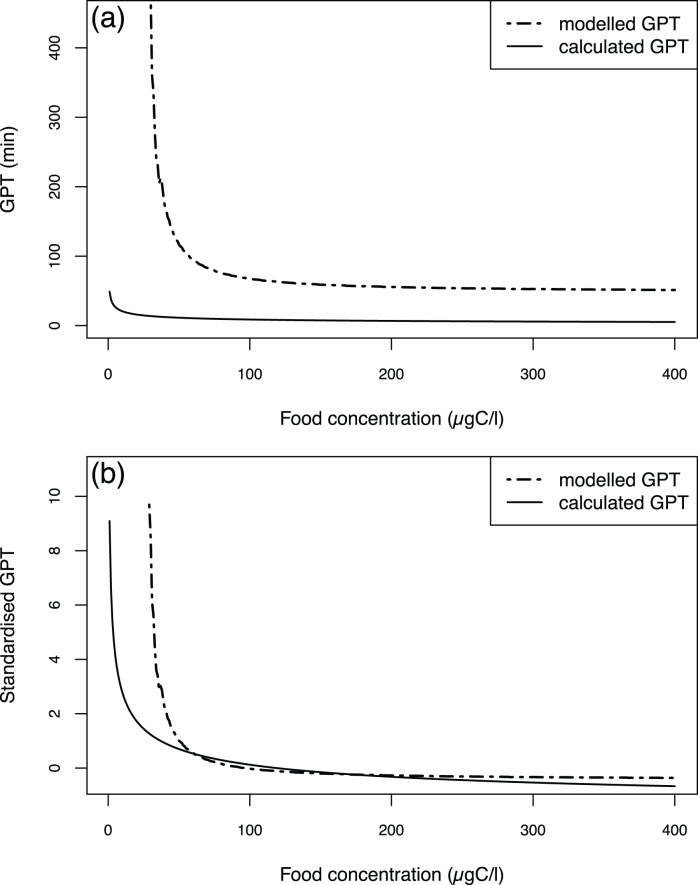
Modelled GPT *vs.* calculated GPT as function of the environmental food concentration (from 0 to 400 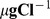). Non-standardised values (a); standardised values (b). The calculated GPT is from López-Urrutia *et al.* [13]. The standardised modelled and calculated GPT are computed by subtracting the overall mean from each GPT value and dividing the result by the overall standard deviation.

**Figure 5:**
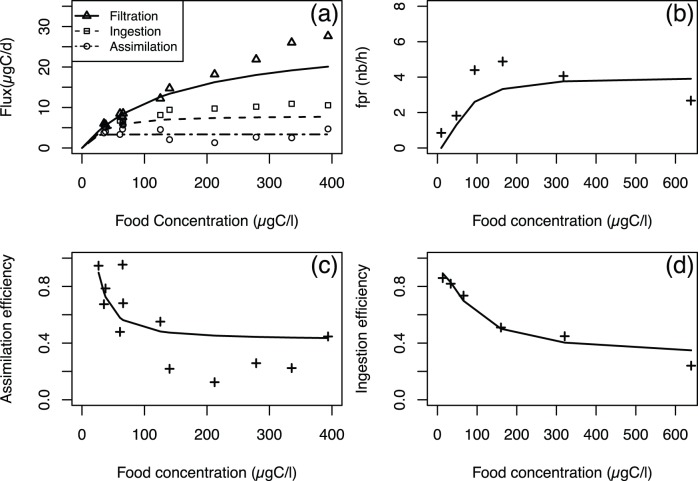
Model outputs (lines) versus experimental data (points) used for parameter estimation. Modelled filtration, ingestion and assimilation rates versus data from [35] (a); modelled faecal pellet rate production versus data from [29] (b); modelled assimilation efficiency versus data from [35] (c); modelled ingestion efficiency data from [27] (d).

**Figure 6:**
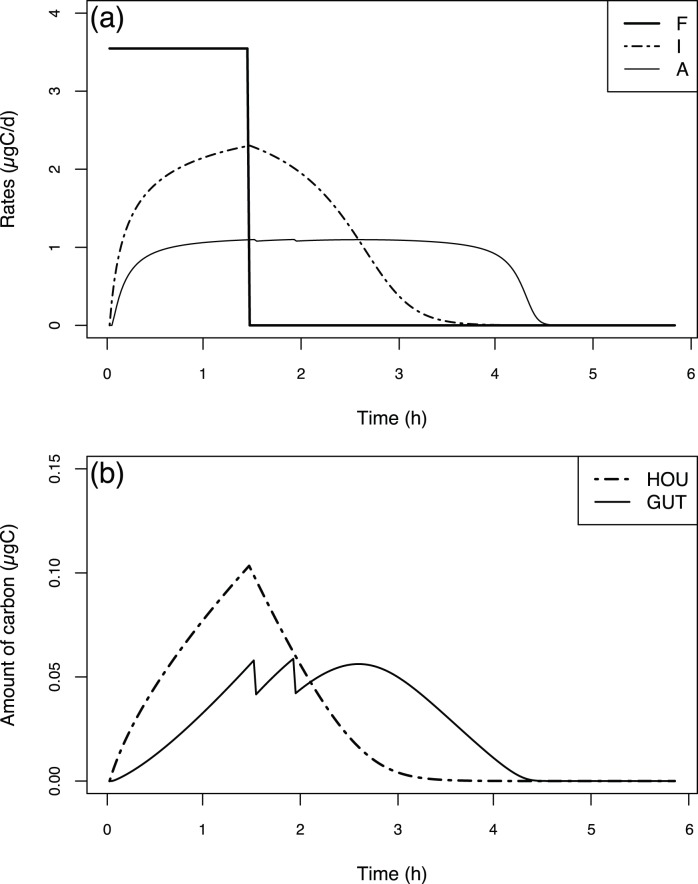
Fluxes and state variables dynamics during simulation with an alimentary interruption occurring at half of the house lifespan (1.44 h). Rates of filtration (F), ingestion (I) and assimilation (A) in 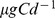 (a); carbon mass of the house contents (HOU) and carbon mass of the gut contents (GUT)(b) in 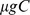. Run performed at a temperature of 15°C and an initial food concentration of 100 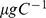 over four house cycles for an individual organism of 1 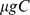.

**Figure 7:**
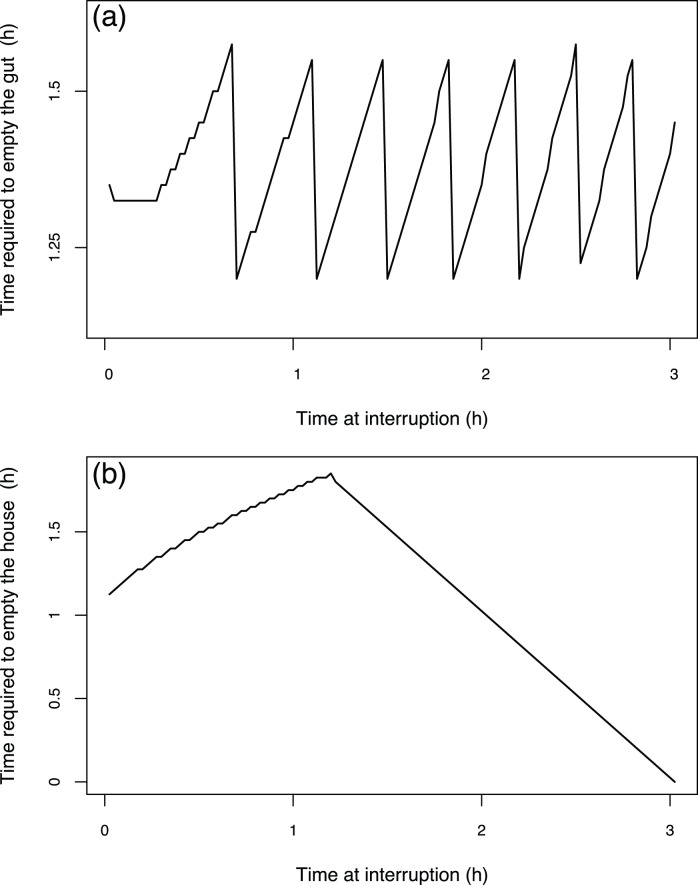
The time required to empty a compartment as function of the moment when alimentary interruption starts during the house lifetime. Times required to empty the gut (a) and the house (b). Simulations with an initial food concentration of 100 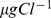 and a temperature of 15°C, and during which alimentary interruptions occurred at different moments of a house lifespan.

**Figure 8:**
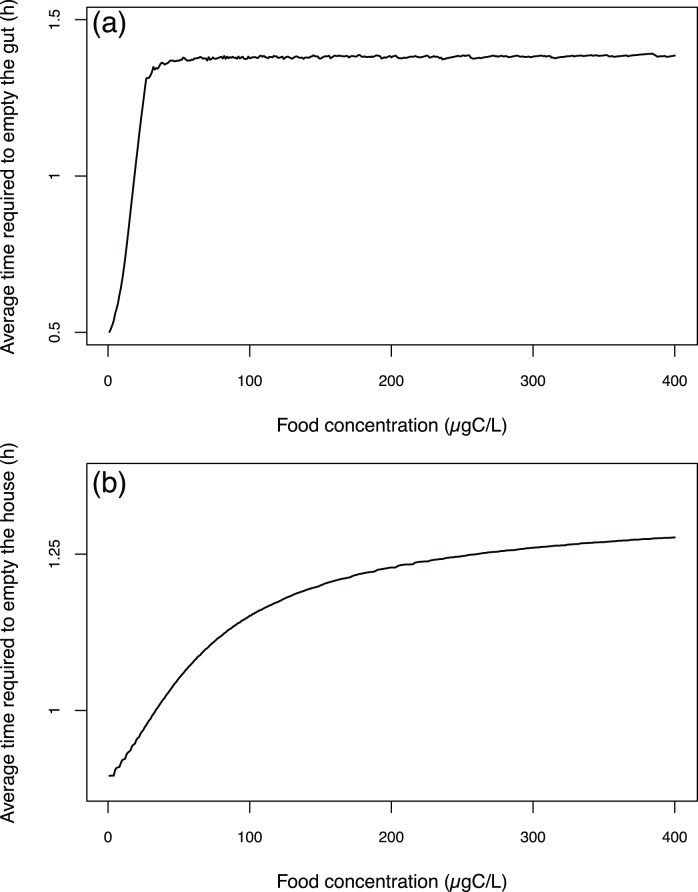
Average time required to empty a compartment as function of the environmental food concentration (from 0 to 400 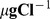). Average times required to empty the gut (a) and the house (b). For each food concentration value, the resulting average emptying time is the average emptying time for simulations during which alimentary interruptions occurred at different moments of a house lifespan.

- Simulations over four house cycles at a fixed environmental temperature (15°C): at a constant food concentration level of 100 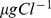 (figure 2); or over a large range of food concentrations to estimate the house carbon accumulation rate (figure 3) and the Gut Passage Time (GPT, figure 4).
- Calibration simulations at a fixed temperature condition and over a large range of food conditions (figure 5).
- To simulate starvation, we ran simulations over one house cycle using initial fixed conditions (T = 15°C and food equal to 100 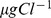) during which an alimentary interruption occurred at half the house lifespan (figure 6), or at different times during the house lifespan (figure 7).
- The same simulations were carried out as detailed above, but at different initial food concentration levels (from 0 to 400 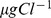), to estimate the average times required to empty the gut and the house (figure 8).

The time required to empty the house is calculated from the time when the food availability is set to zero to the moment when the house content is less than one per cent of its initial value (at food shortage). To calculate the time required to empty the gut, we set the gut initial value to 0.05 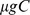 and the house content to zero when alimentary interruption occurs. It is then calculated in the same manner as the time required to empty the house.

From the simulated Defecation Interval time (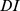) deduced from the faecal pellet production rate, we calculated the modelled GPT, using the equation 8 as developed by López-Urrutia *et al.* [13]. We compared those values with the empirical relationship established from equation 9, which was extracted from figure 6A of López-Urrutia *et al.* [13]. To enable this comparison, we standardised the modelled and calculated GPT from 0 to 400 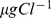, by subtracting the overall mean from each GPT value and dividing the result by the overall standard deviation.
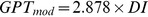
(8)

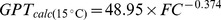
(9)


## Results

### 1 Standard Behaviour of the Model: Simulations at Constant Food Concentrations

Figure 2 presents the model behaviour over a twelve-hour simulation at a constant food concentration (100 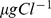) and temperature (15°C) for a 1 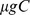 organism. The flux dynamics (filtration, ingestion and assimilation rates) are shown in figure 2a, and state variable dynamics (HOU and GUT) are shown in figure 2b.

Because the food concentration, temperature and body weight are constant, the filtration rate (F) also remains constant, and its value is 3.55 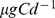. The temporal dynamics of the ingestion rate (I) shows regular cycles at a constant interval time (2.91 hours, see equation 4) linked to the house lifetime. With each new house, the ingestion starts from 0 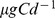. After that, it quasi-instantaneously reaches a value near 2 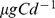, and then it increases with a hyperbolic trend to a plateau of approximately 2.5 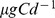. The assimilation rate (A) is almost constant (approximately 1 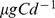), with very small oscillations whose frequency corresponds to faecal pellet production (see below). Because the initial value of the gut was set to 0, the initial assimilation rate starts from 0.

During a house life cycle, the house content (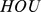) increases in a quasi-linear fashion; the content results from the balance between the filtration and ingestion rates, which is nearly constant a few minutes after house deployment. During the house cycle, the house content varies from 0 to 0.17 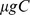. The gut content (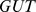) exhibits oscillations, with nearly constant amplitude and frequency, that are linked to the faecal pellet production. Above a maximum gut value (at least 0.059 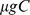 for a 1 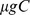 organism, see equations 6 & 7), a faecal pellet is produced, and the gut loses the corresponding weight (0.0175 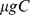 for a 1 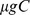 organism, see equation 6). Between two faecal pellet production times, the gut content increases almost linearly, as the difference between the ingestion and assimilation rates is quasi-constant. Because the initial gut content value was set to 0, the first faecal pellet production takes almost 90 minutes.

### 2 Model Validation and Calibration

#### 2.1 House carbon accumulation rate and gut passage time

The house carbon accumulation rate can be calculated from the model for different food concentration levels (figure 3). Its variation pattern plotted against the food concentration describes a sigmoid curve; first, it slowly increases, and then it follows a Michaelis-Menten curve shape that reaches a saturation plateau at a value that depends on the organism weight. For a 3.97 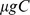 individual, the predicted rate can be compared with experimental data extracted from Acuña *et al.* [27]. At low food concentrations (below 30 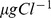), the model accurately simulates the rate of carbon trapped within the house, whereas it overestimates this rate at higher food concentration levels.

An estimation of the GPT can be extracted from the simulated defecation interval times (see equation 8). Faecal pellet production only starts at a food concentration of 30 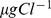 (see below) in our model simulations, and the corresponding modelled GPT is approximately 460 min (figure 4a). After that, the modelled GPT sharply drops below 100 min at 60 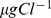 and reaches a plateau of approximately 50 min at 150 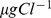 because the faecal pellet production also reaches a plateau at this food concentration level (see below). The calculated GPT from equation 9 presents lower values and a different pattern. The value first decreases from 50 to 15 minutes between 0 and 30 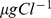 and then decreases more slowly to reach 6 minutes at 400 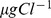. Figure 4b shows the standardised values of the modelled and calculated GPT to compare their variation pattern (see below). The calculated GPT presents a strong decrease between 0 and 30 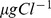 followed by a slower decrease with increasing food concentration. The prediction for the standardised GPT does not fully reproduce the same pattern. It exhibits a strong decrease of the same amplitude between 30 and 60 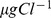, beyond which it saturates.

#### 2.2 Simulations used for parameter estimation

The chosen set of parameters (table 1) is the one that reduces the distance between model outputs and experimental data as shown in figure 5. For each food concentration, the value of the modelled faecal pellet production rate and the values of the modelled filtration, ingestion and assimilation rates, are expressed by using the average process values over a simulation.

The simulated ingestion rate (figure 5a) rapidly increases to reach a plateau around the value of 7 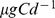 at 130 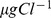, and the half-saturation coefficient regarding the environmental food concentration is approximately 28 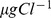. The simulated assimilation rate rapidly reaches a plateau value of 3 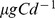 at a 30 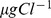 food concentration, and the half-saturation coefficient with respect to the environmental food concentration is approximately 15 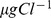. The modelled ingestion rate model appears to have been underestimated. Nevertheless, it is consistent with the data as it reproduces the increasing pattern followed by a saturation plateau. The assimilation rate data seem to be independent of the environmental food concentration. The model captures this behaviour as it predicts a constant assimilation rate within the range of measured values.

The simulated faecal pellet production rate (figure 5b) rapidly increases from 0 to 160 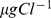 to reach a saturation plateau at 3.5 faecal pellets per hour. The data from Selander *et al.* [29] also present a rapid increase from a food concentration of 0 to 160 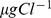, which is accurately reflected by the model, followed by a maximum value of 5 faecal pellets per hour at almost 160 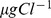. Beyond this concentration, the data present a decrease along increasing food concentrations that is not reproduced by the model.

The assimilation efficiency data present a rapid decrease between 0 and 130 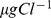 and reach a plateau at approximately 0.25 at this food concentration value (figure 5c). The simulated assimilation efficiencies also present a rapid decrease between 0 and 130 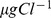 followed by a plateau at 0.4.Therefore, the data pattern is accurately reproduced by the model, although the modelled decrease appears to be excessively fast and not sufficiently strong.

The ingestion efficiency data (from Acuña *et al.* [27]) decrease in a non-linear fashion from 0 to 600 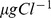 (figure 5d). This decreasing pattern is accurately reproduced by the model, as the simulated ingestion efficiency has the same pattern. Furthermore, the model accurately simulates the ingestion efficiency values in view of the data.

### 3 Hypothesis Testing with the Model: Starvation Simulations

Figure 6 exhibits the model behaviour for a simulation during which an alimentary interruption occurs after 1.45 hours, which corresponds to half the estimated lifespan of a house at 15°C, with an initial food concentration of 100 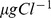 and initial house and gut contents set to zero.

The fluxes and state variable dynamics up until the alimentary interruption are the same as the ones described in figure 2 for a constant food concentration simulation. As soon as the alimentary interruption occurs, the filtration rate instantly falls to zero (figure 6a) because the filtration function depends on the environmental food concentration (see equation 1). Otherwise, the ingestion rate does not immediately fall to zero because the ingestion function does not depend on the environmental food concentration but on the house content (see equation 3). Consequently, this rate falls to zero more than 2 hours later, which corresponds to the house emptying time (figure 6b). The assimilation rate takes a longer time to reach zero because the assimilation function depends on the gut content (see equation 5). It takes about 3 hours after alimentary interruption to reach zero, corresponding to the time required to empty the gut (figure 6b). The decreasing assimilation rate pattern can be divided into two parts. First, the rate starts to decrease slowly when the ingestion rates and house content equal zero. Then, its decrease becomes stronger when the gut content falls below a certain value.

When a house is empty, the ingestion rate equals zero. From this moment, the time required to empty the gut only depends on the assimilation rate, which only depends on the gut content because the body weight and the environmental temperature are constant. Consequently, because the gut content has oscillating dynamics (figure 2b), the time required to empty the gut depends on the moment when the alimentary interruption occurs with respect to the house lifespan (figure 7a). Therefore, the time required to empty the gut fluctuates between minimum and maximum values of approximately 1.2 and 1.55 hours, respectively.

When alimentary interruption occurs, the filtration rate instantaneously falls to zero (figure 6a). Consequently, the duration for emptying the house only depends on the ingestion rate. At a constant environmental food concentration and body weight of the organism, this rate only depends on the house content. As this house content increases during the house cycle, the time required to empty the house depends on the moment when the alimentary interruption occurs in relation to the house cycle (figure 7b). The maximum time required to empty the house is about 1.8 hours when the alimentary interruption occurs about 1.2 hours after the house deployment. After this time, the period required to empty the house decreases because the house is discarded before being completely emptied.

The average times required to empty the gut or the house at a fixed food concentration are the average values of the different possible times (because an emptying time depends on the moment when the alimentary interruption occurs) at this fixed food concentration (figure 8). The average time required to empty the gut below a food concentration of 30 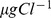 should not be taken into consideration because the model poorly represents the gut throughput dynamics below this value. Above this value, the average time required to empty the gut reaches a plateau of 1.3 hours (figure 8a). The average time required to empty the house quasi-increases with a Michaelis-Menten shape from 0.8 to 1.27 hours at 400 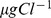 (figure 8b). The variation pattern plotted against the food concentration can be related to the simulated rate of carbon accumulation presented above in figure 3. The time required to empty a house increases with its carbon content.

## Discussion

The proposed formulations for the ingestion and assimilation processes include two state variables, which represent the carbon mass of the house and gut contents. The simulated time-scales of those two variables are difficult to validate because no direct measurements are available. However, indirect estimates are possible from scarce data and will be discussed below. Because the filtration function was extracted from the literature, it will not be discussed, nor will the body weight and temperature functions associated with this function.

### 1 House Content Representation and Ingestion Process

We argue that our predictions for maximum food intake (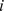, table 1) and half-saturation coefficient (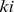, table 1) for ingestion should not be directly compared to published values because they are both a function of the carbon mass of the house content (a state variable). Nonetheless, we computed a mean ingestion rate at 200 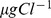 for three different studies and found values at the same order of magnitude for those observed at three different temperatures and body sizes (table 4).

**Table 4:** Comparison of the maximum food intake for ingestion.

Study	Temperature	Trunk length	Body weight	Measured value	Modelled value
[30]	10°C	690 *μm*	4.93 *μgC*	0.164±0.026 *μgCind* ^–1^ *h* ^–1^	0.319 *μgCind* ^–1^ *h* ^–1^
[27]	15°C	637 *μm*	3.97 *μgC*	0.198±0.027 *μgCind* ^–1^ *h* ^–1^	0.327 *μgCind* ^–1^ *h* ^–1^
[29]	20°C	350 *μm*	0.62 *μgC*	0.113 *μgCind* ^–1^ *h* ^–1^	0.080 *μgCind* ^–1^ *h* ^–1^

Comparison of the maximum food intake for ingestion between experimental data and model outputs. weights are obtained using the trunk length to total body length equation from Acuña *et al.* [27] and the body length to carbon weight equation from Lombard *et al.* [35]. For Selander *et al.* [29], the measured value is obtained from the measured value of 14,065 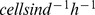 and converted using a carbon weight per cell of 8 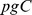.

The half-saturation coefficient quantifies the ways in which the different biological structures of the mouth and foregut (pharyngeal filter and spiracles) limit mass transfer between the house and the gut. In other models which directly relate the ingestion rate to environmental food, the half-saturation coefficient also encompasses limiting effects for structures involved in the filtration process (tail beating frequency, entrance and concentrating filters). The half-saturation coefficient reported in our model is expressed with regards to the carbon mass of the house contents, and there are no equivalent terms in the literature. Its value (0.028 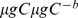, 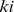 in table 1) is low relative to the dynamics of the carbon mass of the house content (see figure 2b) for a 1 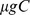 organism. Consequently, the ingestion process saturates at the beginning of the house lifetime and induces carbon accumulation within the house even at low food concentrations (figure 3). It is possible to compare the values of simulated ingestion rates at any time with the external food concentration (figure 5a). Our model simulations suggest a half-saturation coefficient of the ingestion process related to the environmental food concentration of approximately 28 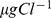. This value is consistent with those reported in Acuña *et al.* [27] and Selander *et al.* [29] (38.9±21.1 and 27 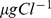, respectively), which were measured under similar food quality and temperature conditions. Regardless of the organism size, the value of the half-saturation coefficient in relation to the environmental food concentration does not change (equation 8, appendix S1).

In the present study, the formulation of the half-saturation coefficient is dependent on the body weight of the organism. This formulation ensures that ingestion efficiency remains constant regardless of the body weight of the organism. *Oikopleura dioica* selects a certain size class of particle thanks to a system of filters (inlet filter, food concentrating filter and pharyngeal filter) with different mesh sizes; because the filter mesh size depends on the organism size (at least for the inlet and the pharyngeal filter, [19]), the selected size class depends on the organism size. Firstly, the inlet filter prevents excessively large particles from entering the house; then, the concentrating filter retains particles larger than its mesh size within the house; and finally, the pharyngeal filter retains a size class of particles between the limits defined by the two preceding filters, as it has coarser pores than the concentrating filter [19]. The particles that are not retained by the pharyngeal filter flow through the spiracles to the house exit chamber [4], [19]. The experimental filtration measurements take all of this information into account by measuring what is removed from the water. Consequently, particles remaining within the house approximately represent the amount of ingestible particles in a targeted size class. Regardless of the size class selected, if the filtration and ingestion intensities are similarly affected by the size of the organism, the proportion of the ingestible particles, *i.e.*, the carbon mass of the house contents, which are actually ingested remains constant regardless of the organism size. Consequently, the carbon mass of the house contents of an organism, or its house carbon accumulation rate, remains constant regardless of the organism size. Nevertheless, a limitation to this assumption should be addressed. The experiments used in this work have mono-algal dietary conditions. Moreover, the diameter of the algal species used is comprised in a size range that almost remains ingested and filtered with the same efficiency whatever the organism size [19]. Under these conditions, changes in the ingestion and filtration efficiencies with the size of the organism do not occur. Nevertheless, this food condition with no differential particle size selection for different organism sizes provides a good context for better understanding the mechanisms involved in the energy uptake processes.

The modelled carbon mass of the house contents can be addressed with Acuña *et al*.’s [27] experimental measurements at different food concentrations for the carbon accumulation rate (figure 3), which are in accordance with more recent measurements from Troedsson *et al.* [38]. The model reproduces these measurements correctly below the 30 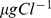 food concentration value and overestimates them above that value. This overestimation is larger with higher food concentrations and can be explained as follows. When houses are discarded, the carbon within them is rapidly diffused outside, as the water-flow created by the filtration process which had kept the particles inside no longer exists [39]. Consequently, the experimental measurements are minimum estimations of the house contents. In conclusion, we can assume that the model estimations for the carbon mass of the house contents are correct with respect to Acuña *et al*.’s [27] data. The results showed in figure 3 exhibit a sigmoid curve. The explanation of this observation is as follows. The half-saturation coefficient of the mean ingestion (figure 5a) is about 28 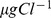 and corresponds to the first point of inflection. The second inflection point corresponds to the half saturation of the filtration function (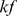 = 150, table 1). The consequence is that the saturation value depends on the weight of the organism, which is relevant to the fact that a smaller organism has a smaller house and therefore a lower carbon mass content. Nevertheless, the carbon mass of the house contents for two different-sized organisms are the same in relation to their weight, with the same dependence on the power function (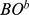) that influences the filtration and ingestion rates (equation 10, appendix S1).

### 2 Faecal Pellet Production and the Assimilation Process

The way we formulate the carbon mass dynamics of the gut contents is consistent with experimental observations [13], [34] and allows us to make mechanistically inspired predictions for faecal pellet production. To form a faecal pellet, *Oikopleura dioica* packs the whole foregut (*i.e.*, the stomach), which is thus emptied each time a faecal pellet is formed [13]. Therefore, we assume that the faecal pellet transit in the digestive tract of the organism is only due to the gut filling in the present model. No faecal pellet transit by cilia beating along the digestive tract is considered; if ingestion stops, no more faecal pellets will be produced.

With respect to the gut content, López-Urrutia *et al.* [20] proposed a mathematical relationship elaborated from several *in situ* data, which allowed them to calculate the total carbon mass of the gut content from the weight of the organism as indicated in their table 3. From their equation, we can estimate that the gut content should comprise between 0.072 and 0.081 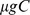 for an organism weight of 1 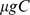, whereas our model simulates a maximum value of 0.059 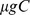. To obtain a similar range value in our model, we must change the value of the parameter 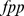 from 0.0175 to 0.03, which is exactly the value estimated from the gut content data of López-Urrutia *et al.* [31]. Indeed, in their table 3, they calculated a gut food-volume of 286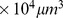 for an organism measuring 607 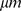 (trunk length). If we estimate the weight of this gut filled with spherical particles of 5 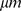 in diameter and containing 8 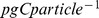 (as in *Isochrysis galbana*), we find a value of 0.35 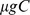 (*i.e.*, a faecal pellet weight of 0.10) for an organism weight of 3.4 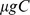 (by using trunk length to total body length equation from Acuña *et al.* [27] and body length to carbon weight from Lombard *et al.* [35]). The corresponding 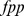 value obtained in this manner is 0.0302.

In examining the faecal pellet production rate, if we compare the model output with data from Selander *et al.* [29], we notice the following two differences (figure 5b): first, the modelled increase up to an environmental food concentration of 160 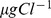 is too low; second, the decrease observed beyond that point is not reproduced by the model. With respect to the first issue, because the assimilation rate is accurately simulated according to the data (figure 5a, see below), we could infer that the estimated faecal pellet weight is too high, resulting in a lower production rate. However, this assessment is not supported by other published data, so the estimated faecal pellet weight of our model cannot be considered to be too high. Thus, a possible explanation might be the excessively low simulated ingestion rate, as supported by figure 5a. With respect to the second issue, we offer two possible explanations; the first one regards the existence of the decrease. In fact, this finding was not actually observed by López-Urrutia *et al.* [13], as they reported a GPT that decreases with increasing food concentration (figure 4b). The second explanation is that the model fails to consider an unknown mechanism that acts at high food concentrations and either increases the faecal pellet weight or reduces the assimilation efficiency. The latter could be supported by figure 5c, which demonstrates that the model estimates high assimilation efficiencies compared with the data at high food concentrations.

Another point that should be discussed is that the model does not simulate the faecal pellet production below a food concentration of 30 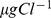 (figure 4). Indeed, when we compared the modelled gut passage time as estimated from the defecation interval (equation 8) with published data, we noticed two problems. First, because there is no faecal pellet production below 30 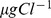, a lag is observed between the data and the model. Below this food concentration level, the gut content increase is too small and will never be filled because the gut content is affected by every house replacement (figure 2b). Second, the model output appears not to reproduce the estimated GPT from the equation as shown in figure 6A of López-Urrutia *et al.* [13] (figure 4a). Indeed, the faecal pellet production rate data from Selander *et al.* [29] and the GPT estimation from López-Urrutia *et al.* [13] are not compatible. The defecation interval deduced from Selander *et al.*’s [29] data is 4 to 10 times higher than the one deduced from the López-Urrutia *et al.* GPT estimation [13], implying that the gut passage time is longer in the former study. This result does explain the discrepancy between the modelled GPT, which is obtained from the defecation interval using equation 9, and the data from Lopez-Urrutia *et al.* [13], as the parameters that act on the faecal pellet production were estimated from the rate of faecal pellet production data from Selander *et al.* [29]. Nevertheless, the patterns of the simulated and calculated GPTs versus the food concentration are similar (figure 4b), meaning that the major mechanism influencing faecal pellet production is taken into account by the model.

In any case, the inconsistency of no faecal pellet production below 30 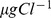 underlines the fact that representing the faecal pellet production using the gut filling process alone is not sufficient. At low food concentration levels, we can assume that faecal pellets are not formed by packing the entire gut content, meaning that the faecal pellets are less compacted and represent a lower proportion of the organism weight than at higher food concentration levels. Therefore, there should be another mechanism that acts on the faecal pellet production at all food concentration levels but becomes a dominant mechanism only at low food concentration levels. Microvillar ciliated cells are widely distributed along the entire digestive tract of *Oikopleura dioica*. The ciliary activity and cytoskeletal microfilaments modulate the diameter of the lumen, allowing the valves to coordinate the unidirectional passage of food from one region to the next [40].

The assimilation rate data from Lombard *et al.* [35] are accurately reproduced by the model (figure 5a & 5c). The half-saturation coefficient is independent of the organism’s body weight (equation 9, appendix S1), and it is also in accordance with those data (15 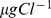). However, we note that few published data on assimilation for *Oikopleura dioica* are available, to our knowledge. The present model calculates a carbon assimilation of 1.09 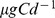 for an organism weight of 1 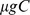 at 15°C. Given this amount of assimilated carbon, the organism grows, maintains its tissues and produces houses. From Sato *et al.* [10], we can estimate (with 7 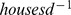 and one house weighing 15.4±4.8% of the organism’s weight) a house accumulated weight production of 1.08±0.336 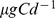. This value is large relative to the amount of assimilated carbon, leading to the argument that the rate of house production may be lower, as measured in Fenaux *et al.* [33]. Another argument could be that discarded houses contain less carbon than newly deployed ones, as reported for *Oikopleura rufescens* in Alldredge *et al.* [41]. Therefore, experimental measurements of assimilation rates may be underestimated because they are calculated from the amount of discarded houses, plus estimations of the organism carbon growth and the carbon loss by respiration. Therefore, in a growth model of this organism, either the carbon weight of a house should be lower than reported by Sato *et al.* [10] because the authors performed their measurements on newly secreted houses, or the assimilation rate should be deliberately overestimated.

### 3 Time Required for Responding to an Environmental Food Fluctuation

*Oikopleura dioica* has some internal structures of reserves [42], but they are low [43]. The house and gut represent reservoirs of ingestible and assimilable food that can be considered as external pools of reserves, which lead to smooth environmental fluctuations in food conditions. When an environmental food depletion occurs, the house is able to compensate during a variable period of time that depends both on the time when the depletion occurs during the house lifespan (figure 7b) and on the food concentration experienced before food depletion (figure 8b). Because our model estimates the house content and ingestion rates in a realistic fashion (see above), we assume that the time required to empty the house is correct. The average time required to empty the house (figure 8b) can be considered as the delay time of an appendicularian population, as the individuals are not all synchronised in their temporal house production dynamics.

When the depletion occurs over a period of time long enough to empty the house, the gut starts to empty and the assimilation is then affected (figure 6). The simulated average time required to empty the gut is constant above a food concentration of 30 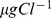 and is lower below that concentration because the gut content is lower. In the present study, the time required to empty the gut is the result of the assimilation only, which is most likely the reason why it is higher than reported in López-Urrutia *et al.* [13].

The organism growth will start to be affected only when the gut is empty and, consequently, the assimilation is affected, or perhaps the growth will be affected a bit later because the organism has a low density of reserves. The time scale required for an organism to respond to a variation in its environmental food condition can be important relative to its life time; consequently, it should be taken into account in the functional response of this organism. Survival experiments, that generate an estimation of the time required to observe the death of an organism, could be an appropriate experiment for investigating whether the response time is as important as suggested by the model outputs.

## Conclusions

The model presented here proposes a new mechanistic formulation of ingestion and assimilation, which are two steps of the feeding process. This model mirrors the physiology of those processes by representing the carbon mass of the house and gut contents. Model outputs are compared with several published data [13], [20], [27]–[30], [35], [38] to discuss different hypotheses.

The new formulation of the ingestion process which is linked to the representation of the carbon mass of the house content enables a realistic estimation of the carbon accumulation rate within the house. This formulation is based on the assumption that, at a constant food concentration, the amount of ingested particles over the amount of particles within the house should remain constant whatever the size of an organism if the filtration and the ingestion intensities of the targeted size class of particles are similarly affected by the size of the organism. Moreover, our study also shows that the house plays the role of an external reserve of ingestible particles, which enables the organism to resist food depletions for a time scale of one hour. Based on this finding, the house can be regarded as a structure that compensates for the low reserve density of these organisms.

The assimilation process is poorly documented in the literature because it is extremely difficult to quantify and, to our knowledge, only a few studies focus on this issue, both for *Oikopleura dioica* [35] and for other appendicularian species [44]. The present work investigates the process of assimilation in combination with faecal pellet production based on literature experiments. Our work highlights the possibility that at environmental food concentrations above 30 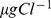, faecal pellets may represent 3% of the total body weight for *Oikopleura dioica*. The model outputs further suggest that below this food level faecal, pellets should represent a smaller fraction of the total body weight. We explain this feature as a shift in how two synergistic processes, responsible for the throughput dynamics of faecal pellets, interact at different food levels. Even if the rate of faecal pellet production is not well represented in the present model below a food concentration of 30 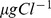, the correct reproduction of ingestion and assimilation rates below this food concentration provides a good estimation of the unassimilated carbon mass through the difference between those two rates.

Appendicularians have a unique feeding method that must be understood at the individual scale if we want to better estimate the impact of a population of appendicularians on pelagic ecosystems. Our model could be used for that purpose, as it accounts for the time required to respond to environmental food fluctuations.

## Supporting Information

Appendix S1 **Weight independence of I/F and A/I.**
(PDF)
