# Supplementary material for: A Mechanistic Individual-Based Model of the Feeding Processes for Oikopleura dioica
Source: PLoS One. 2013 Nov 5;8(11):e78255. doi: 10.1371/journal.pone.0078255 (PMC3818322; doi:10.1371/journal.pone.0078255)
Supplement: Appendix S1 — Weight independence of I/F and A/I. (PDF) [file pone.0078255.s001.pdf]

Weight independence of I/F and A/I

Maxime Vaugeois[1,2,*], Frédéric Diaz[1,2], François Carlotti[1,2]
1 Aix Marseille Université, CNRS/INSU, IRD, Mediterranean Institute of Oceanography (MIO), UM 110, 13288 Marseille, France
2 Université de Toulon, CNRS/INSU, IRD, Mediterranean Institute of Oceanography (MIO), UM 110, 83957 La Garde, France
∗ E-mail: maxime.vaugeois@univ-amu.fr

We know HOU and GUT as fallows:

$$\frac{\mathrm{d}HOU}{\mathrm{d}t} = F - I \tag{1}$$

$$\frac{\mathrm{d}GUT}{\mathrm{d}t} = I - A \tag{2}$$

With the following:

$$F = f \times BO^b \times \frac{FC}{FC + kf} \tag{3}$$

$$I = i \times BO^b \times \frac{HOU}{HOU + ki \times BO^b} \tag{4}$$

$$A = a \times BO^b \times \frac{GUT}{GUT + ka \times BO^b} \tag{5}$$

We pose u and v as follows:

$$u = \frac{HOU}{BO^b} \tag{6}$$

$$v = \frac{GUT}{BO^b} \tag{7}$$

So:

$$I = \frac{i \times u \times BO^b}{u + ki} \tag{8}$$

$$A = \frac{a \times v \times BO^b}{v + ka} \tag{9}$$

And so, when FC is constant, equations 1 and 2 can be written as follows:

$$\frac{\mathrm{d}HOU}{\mathrm{d}t} = BO^b \times \left[C - \frac{i \times u}{u + ki}\right] \tag{10}$$

$$\frac{\mathrm{d}GUT}{\mathrm{d}t} = BO^b \times \left[\frac{i \times u}{u + ki} - \frac{a \times v}{v + ka}\right] \tag{11}$$

with the constant C as follows:

$$C = f \times \frac{FC}{FC + kf} \tag{12}$$

So:

$$\frac{\mathrm{d}u}{\mathrm{d}t} = \frac{1}{BO^b} \times \frac{\mathrm{d}HOU}{\mathrm{d}t} \tag{13}$$

$$\frac{\mathrm{d}u}{\mathrm{d}t} = C - \frac{i \times u}{u + ki} \tag{14}$$

$$\frac{\mathrm{d}v}{\mathrm{d}t} = \frac{1}{BO^b} \times \frac{\mathrm{d}GUT}{\mathrm{d}t} \tag{15}$$

$$\frac{\mathrm{d}v}{\mathrm{d}t} = \frac{i \times u}{u + ki} - \frac{a \times v}{v + ka} \tag{16}$$

Because those differential equations do not explicitly depend on BO, solutions u(t) and v(t) cannot depend on BO, so

$$\frac{\partial u}{\partial BO} = 0 \qquad and \qquad \frac{\partial v}{\partial BO} = 0 \tag{17}$$

I/F and A/I can be written as follows:

$$\frac{I}{F} = \frac{\frac{i \times u}{u+ki}}{C} \tag{18}$$

$$\frac{A}{I} = \frac{\frac{a \times v}{v+ka}}{\frac{i \times u}{u+ki}} \tag{19}$$

Because those equations only depend on u and v,

$$\frac{\partial \frac{I}{F}}{\partial BO} = 0 \tag{20}$$

$$\frac{\partial \frac{A}{I}}{\partial BO} = 0 \tag{21}$$
